# Impact of smart and digital transformation on pharmacy intravenous admixture services in a tertiary-grade A hospital

**DOI:** 10.3389/fphar.2026.1798701

**Published:** 2026-08-26

**Authors:** Cong-Cong Xu, Shu-Yan Zhang, Rui-Han Yuan, Ji Li

**Affiliations:** Pharmacy Intravenous Admixture Services, Qilu Hospital of Shandong University (Qingdao), Qingdao, Shandong, China

**Keywords:** digitalization, error rate, information collection, intelligent distribution, intelligent labeling, intelligentization, pharmacy intravenous admixture services, work efficiency

## Abstract

**Objective:**

This study aims to assess the impact of smart and digital transformation initiatives on the operational efficiency, personnel allocation, and error rates within the Pharmacy Intravenous Admixture Services (PIVAS) at a tertiary-grade A hospital.

**Methods:**

Key components of the smart and digital transformation implemented in PIVAS were described. Comparative analyses were conducted on work efficiency metrics, staffing distribution, and error rates across two 3-month periods prior to and following the transformation.

**Results:**

Post-transformation, workflow processes were significantly optimized, with the average labeling time per infusion label reduced from 6.19 ± 0.87 s to 3.08 ± 0.43 s. The time required to deliver an equivalent quantity of infusions decreased from 103.12 ± 18.90 min to 69.81 ± 14.87 min. The number of personnel involved in the labeling process was reduced from 6 to 4. Despite an increase of 20 wards, no additional personnel were needed for infusion delivery. The labeling error rate declined from 0.072% ± 0.086% to 0.0084% ± 0.022%, and the delivery error rate decreased from 0.033% ± 0.048% to 0.0073% ± 0.020%.

**Conclusion:**

The intelligent digital transformation has significantly improved operational performance, accelerated label application speed, enhanced delivery efficiency, reduced staffing requirements, and lowed labeling error rates. These improvements support the sustainable development and operational performance of PIVAS in large healthcare institutions.

## 1 Introduction

In recent years, the advancement of smart hospitals facilitated by policy initiatives and technological progress has emerged as a prominent trend within the healthcare sector. The integration of new technologies and operational models, including Internet+ and artificial intelligence, has been instrumental in supporting the development of smart hospital infrastructure (Li, 2025). Pharmacy Intravenous Admixture Services (PIVAS) centralize the preparation of intravenous infusion medications, a task that was previously performed by nursing staff in ward-based, open environments. This process is now conducted within controlled facilities by trained technical personnel, utilizing workstations located in Class 10,000 cleanrooms, with designated areas meeting Class 100 cleanroom standards (Jessurun et al., 2022). As a key clinical unit encompassing drug management, prescription review, sterile compounding, and distribution, the implementation of PIVAS has contributed to a reduction in the workload of frontline clinical nurses and has enhanced occupational safety for healthcare personnel (Tian et al., 2024). Compared to traditional pharmacies, PIVAS is characterized by high workload, susceptibility to errors, and labor-intensive operations, with significant differences in operational procedures compared to oral medication dispensing. With the rapid expansion of PIVAS in China, significant attention has been directed toward the smart and digital transformation of PIVAS in tertiary hospitals, accompanied by a growing body of related research. The primary objective of smart and digital transformation is to replace conventional manual processes with computerized systems and automated equipment, thereby reducing labor demands, minimizing operational errors, and improving the safety of intravenous infusion therapy within hospitals (Wang et al., 2022).

The PIVAS at the study hospital was established in December 2013. In 2024, following the implementation of Phase II operations of the hospital, the scale of PIVAS operations expanded, with the number of serviced wards increasing from 20 to 40. This expansion enabled centralized compounding of approximately 3,500 bags of intravenous infusion medications per day. The service was responsible for the preparation of both standing order and pro re nata (PRN) intravenous infusions, with a total of nine batches delivered to wards daily. The PIVAS workflow comprised prescription review, drug dispensing, labeling, compounding, double-checking of finished infusion products, packaging, and delivery. After the opening of Phase II, a smart and digital transformation was implemented to further improve operational efficiency, reduce error rates, and enhance information traceability within PIVAS. This transformation included the deployment of the SmartPrinter V5.0-TP-12 intelligent labeling and sorting system, upgrades to the packaging information collection system, and the introduction of a robotic logistics distribution system. This renovation has improved operational efficiency, reduced required staffing, enhanced operational accuracy, and enabled full information traceability, thereby enhancing overall PIVAS management efficiency. The implementation of the smart and digital transformation of PIVAS is described below.

## 2 Materials and methods

### 2.1 Data sources

Operational data from two separate 3-month periods prior to and following the implementation of the smart and digital transformation of PIVAS at the hospital were collected and used to define the pre-transformation and post-transformation groups. The dataset included labeling time, delivery time of finished infusion products, the number of errors in labeling and delivery processes, and staffing allocation. No changes were made to the number or composition of personnel between the two periods. In terms of workload, following the implementation of Phase II of the hospital in 2024, an increase of approximately 1,000 infusion bags per day was observed in the post-transformation period compared with the pre-transformation period. During the observation period, no significant PIVAS-specific quality improvement initiatives, personnel configuration policy adjustments, or regulatory changes were implemented that could reasonably explain the observed improvements in magnitude and timing.

### 2.2 Methods

In 2024, the hospital’s PIVAS entered Phase II, during which a smart and digital transformation was implemented. This transformation included the integration of an intelligent labeling system, a robotic logistics distribution system, and an upgraded packaging information collection system. The information workflow within PIVAS was restructured to establish interconnection and interoperability between the Hospital Information System (HIS) and intelligent equipment systems, thereby ensuring a closed-loop process for information transmission.

#### 2.2.1 PIVAS process reengineering

The PIVAS workflow is characterized by a high degree of interconnectivity. In the traditional process, the extent of smart and digital integration was limited, which hindered data traceability. Despite substantial labor input, error rates remained elevated, thereby increasing medication-safety risks for patients (Yang et al., 2023). Based on the smart and digital transformation model, the workflow of PIVAS at the hospital was reengineered. The complete intravenous medication workflow comprised the following stages: Physician issues medical orders → Information transmission → Allocation of infusion batches → Drug dispensing and accounting → Intelligent labeling → Label verification → Drug preparation → Final infusion product review → Sorting and scanning → Packaging → Robot delivery → Ward reception. Order entry, information transmission, and drug dispensing followed standardized hospital-wide procedures and were not modified during the study. The duration of these stages remained stable throughout. The intervention focused on labeling, secondary verification, and ward distribution, and the primary outcomes assessed were time efficiency and error rates during these phases. Following the process reengineering, corresponding standard operating procedures, job responsibility frameworks, and performance-based reward and disciplinary systems were established to ensure adherence to the revised processes and regulatory compliance. Comprehensive training and assessments were conducted for all personnel involved (Chen J. et al., 2021). Intelligent equipment engineers provided on-site, one-on-one training for all staff batches, ensuring comprehensive coverage and mastery. Standardized instructional videos were made available for ongoing reference, and concise operating procedures were posted on the equipment to minimize operational errors and support efficient use. Post-training assessments were administered to confirm competency across all staff.

#### 2.2.2 Intelligent labeling system

The intelligent labeling system used in our hospital is the SmartPrinter 5.0-TP-12 (Suzhou Weijie Pharmaceutical Technology Co., Ltd., Suzhou, China). Prescription data are transmitted through a standardized closed-loop workflow. Clinical physicians enter electronic prescriptions via the Hospital Information System (HIS), which are reviewed and approved by a licensed pharmacist. Approved intravenous medication data are then transferred through the hospital intranet to the PIVAS management system, where are consolidated and label information is automatically sent to the labeling system for batch printing. Operators first printed a drug dispensing summary list based on the batch-specific drug information displayed by the system. The drug information on the summary list was fully consistent with that on the infusion labels, and drugs requiring compounding were clearly differentiated from those that did not, thereby streamlining the drug dispensing process. Labeling and drug dispensing procedures were conducted concurrently, functioning independently and without mutual interference.

During the intelligent labeling process, the system simultaneously performed identification, verification, printing, and label application (Deng et al., 2024). In cases where a mismatch between the solvent and the infusion label was found, the system automatically halted transmission and did not proceed with labeling the incorrectly positioned solvent. When labeling was completed, the conveyor belt transferred the labeled solvent to a designated collection box, sorted according to preset compartment configurations.

During operation, when a solvent replacement was required, voice prompts were issued by the system. Operators replaced the corresponding solvent and resumed the printing and labeling process by following both the auditory instructions and the solvent information displayed on the main control panel. To facilitate rapid identification and accurate replacement, different types and specifications of solvents were marked using distinct font colors on the control panel interface. The font color displayed by the system corresponded with that printed on the solvent packaging. Each label included a unique serial number printed in the lower right corner, which served as a reference for subsequent verification.

**FIGURE 1:**
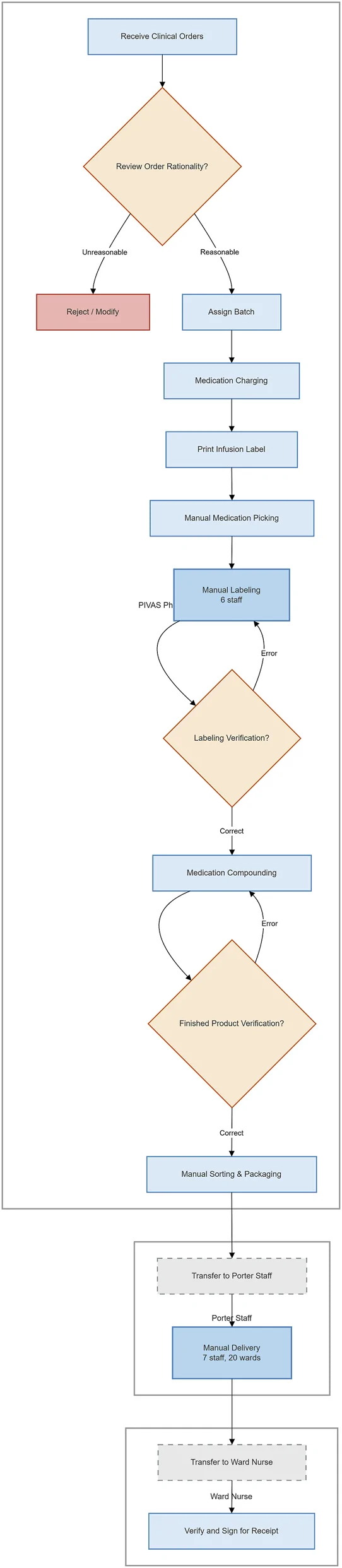
Workflow prior to implementing the intelligent labeling system.

**FIGURE 2:**
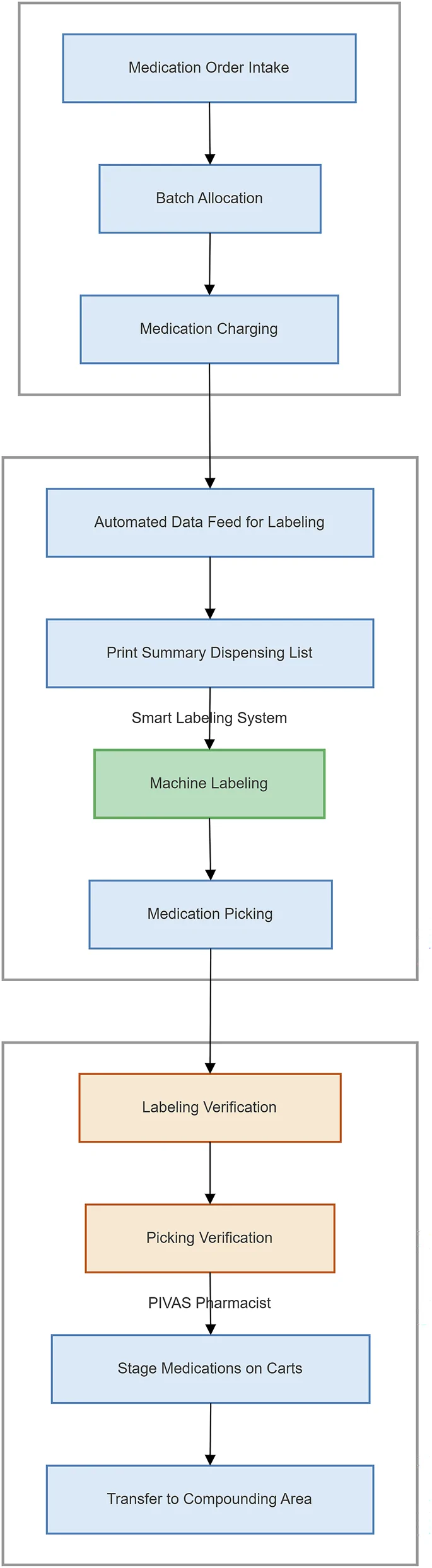
Workflow after implementing the intelligent labeling system.

Additionally, all labeling operation data could be retrieved for statistical and traceability purposes. Query parameters included ward, patient name, bed number, active ingredient, solvent, and batch number, enabling review of infusion label status, printer used, and the specific printing time. Full traceability of the labeling process was thereby achieved. Workflows prior to and following implementation of the intelligent labeling system are depicted in Figures 1,2.

#### 2.2.3 Packaging information collection system upgrade

The sorting and packaging stage for compounded finished infusion products had previously been identified as a high-incidence point for errors within the hospital’s PIVAS (Chen J. et al., 2021). In 2021, a PIVAS intelligent information collection system was developed within the hospital’s HIS. Through this system, barcode scanning verification was required for all compounded infusion products prior to their entry into the packaging phase. Only after successful verification could products proceed. The system displayed relevant drug information, patient details, and packaging data, and provided summary statistics. As part of the recent smart and digital transformation, the original packaging information collection system was upgraded. A “one-click completion” function was integrated into the system module. When the finished infusion products for all wards were scanned, activation of the “one-click completion” function generated summary QR codes for each ward. These were automatically ordered based on inpatient building location, from highest to lowest, and printed accordingly. This process replaced the earlier method, which required manual generation and printing of summary codes for each department, thereby improving operational efficiency and reducing processing time.

Additionally, a module was introduced on the ward-side interface to enable queries of intravenous medication information. Through this module, ward nursing staff were able to access patient-specific data, infusion product details, and PIVAS packaging records. This allowed for direct retrieval of information regarding finished infusion products without requiring direct communication with PIVAS personnel. The upgrade eliminated information gaps between clinical departments and PIVAS, facilitated more efficient information sharing, and contributed to a reduction in the workload of ward nurses.

#### 2.2.4 Intelligent delivery robot

Under the traditional model for delivering finished infusion products, PIVAS staff transferred packaged infusion products to support personnel according to ward assignments. These products were then delivered to each ward using logistics carts and handed over to ward nurses. This manual delivery model was associated with several limitations, including incorrect retrieval, insufficient delivery personnel, reduced efficiency due to high labor intensity, and limited traceability.

To address these issues, intelligent delivery robots were introduced following the implementation of Phase II of the hospital. These robots were adopted to replace a substantial portion of the manual delivery process for finished infusion products. The intelligent delivery robot used in this study was the Zhisaila C2 (Guangzhou Sait Intelligent Technology Co., Ltd., Guangzhou, China). After packaging by ward, the products were placed by staff into compartmentalized delivery boxes within the robot. Orders were submitted via the operational display screen, specifying the destination department, after which the robot commenced autonomous delivery along preprogrammed routes.

It features a modular design comprising a central robotic unit and interchangeable racks, with each rack containing four box units. This configuration enables the simultaneous delivery of medications to four departments. Each unit has a walking speed of 2.0 m/s, a volume capacity of 75 L per box unit, and a maximum load capacity of 300 kg per shelf. Access to individual box units was restricted; only designated personnel from the corresponding department were able to open their assigned compartment for retrieval or placement of drugs. The robot was equipped with 5G connectivity, enabling autonomous navigation, elevator use, door access, and self-charging. Additionally, the system could record delivery data and generate logistics reports to support operational management. Video surveillance of robot movement and retrieval activity was included to enhance delivery traceability. Through integration with the HIS, robot delivery data were transmitted in real time to ward-level interfaces, enabling seamless information exchange.

Ward nurses were able to access finished infusion product delivery status through computer terminals or Personal Digital Assistants, facilitating increased workflow management efficiency. Given the logistical complexity associated with finished infusion product distribution in PIVAS, a comprehensive information query system enabled timely and accurate tracing of all process stages. This functionality supported targeted adjustments to nursing workflows and contributed to improved operational efficiency among ward nurses (Peng et al., 2023).

#### 2.2.5 System failure emergency response plan

To ensure the operational continuity and manage risks associated with intelligent system failures, a structured emergency response plan was established for each system component.

Smart labeling system: Minor failures, such as solvent identification errors, software crashes, or paper jams, are addressed on-site by duty personnel with remote support from equipment engineers. In the event of a severe failure, manual labeling is initiated immediately: operators use infusion labels printed by the HIS system to complete labeling and verification by conventional methods, ensuring uninterrupted operations. The Information Department and equipment engineers are notified concurrently for remote or on-site repair.

Intelligent delivery robot: In the event of path deviation, hardware failure, or elevator malfunction, manual delivery is activated immediately, with property management staff ensuring finished infusion products reach the ward within the required timeframe. The robot undergoes a self-check at each startup and scheduled maintenance once per month to reduce failure risk.

Packaging information collection system: If data transmission between the HIS system and smart devices is interrupted, manual verification and registration are performed, while the Information Department investigates the cause and synchronizes data upon system restoration.

During the 3-month post-implementation observation period, four minor failures were recorded in the intelligent labeling system: two solvent identification errors and two paper jams, all resolved within 10 min. The delivery robots and HIS packaging information collection system operated without incident. Overall, the failure rate was low and no medication delivery delays occurred.

### 2.3 Outcome measures

Comparisons were conducted between the pre- and post-transformation periods with respect to the following parameters: labeling time per medical order for standing order batches 1 and 2; delivery time of finished infusion products for standing order batch 1; frequency of errors occurring during labeling, packaging, and delivery processes; and the number of personnel allocated to each stage.

### 2.4 Statistical methods

This study employed a before-and-after self-controlled design within the same operational unit (PIVAS), comparing performance across two 3-month periods before and after the smart and digital transformation. The unit of analysis was daily aggregated workflow data, including labeling time, delivery time, and error rate, collected across working days within each 3-month period, yielding paired daily observations between the pre- and post-transformation periods.

All continuous variables were first assessed for normality using the Shapiro-Wilk test (α = 0.05), applied to the paired differences between corresponding pre- and post-transformation observations rather than to the raw daily values. Error rates were calculated as continuous daily percentages (errors per total infusions processed per day) rather than as discrete counts, allowing them to be evaluated using the same normality and testing framework as the other continuous outcomes. Normality results confirmed that the paired differences for all outcome indicators, label application time, delivery time, and error rate, were normally distributed, satisfying the assumption required for parametric paired testing.

Because each post-transformation observation corresponds to the same work process, staffing structure, and operational unit as its pre-transformation counterpart, differing only in the presence or absence of the digital transformation intervention, the resulting data are dependent (paired) rather than independent. Paired-sample t-tests were therefore applied to all comparisons of efficiency and quality indicators before and after process improvement. Statistical significance was set at P < 0.05.

## 3 Results

### 3.1 Comparison of work efficiency prior to and following transformation

Following the implementation of the smart and digital transformation, a significant reduction in the time required for labeling and delivery processes was observed, despite no changes in the number or structure of PIVAS personnel and an increased workload. This demonstrated a notable improvement in overall work efficiency. The differences between the pre- and post-transformation periods were statistically significant (*p* < 0.05), as presented in Table 1.

**TABLE 1:** Comparison of labeling and delivery work efficiency prior to and following transformation (*n* = 3,000).

Period	Labeling time per label (s)	Delivery time for same workload of infusions (min)
Pre-transformation	6.19 ± 0.87	103.12 ± 18.90
Post-transformation	3.08 ± 0.43*	69.81 ± 14.87*
*t*	30.882	13.288
*p*	0.000	0.000

Measurement data are expressed as mean ± standard deviation (x̄ ± s). Comparison before and after intervention was performed using the paired-sample t-test.

* P < 0.001 indicates significant difference compared to pre-intervention levels.

### 3.2 Comparison of staffing allocation prior to and following transformation

Prior to the transformation, the standing order labeling and double-check procedures required six personnel. Following the implementation of the intelligent labeling system, and despite a daily increase of approximately 1,000 infusion bags, all labeling tasks were completed by one staff member operating the labeling machine, while three additional personnel were assigned to conduct the labeling double-checks. This resulted in a reduction in total staffing from 6 to 4. In the delivery process, PIVAS previously served 20 clinical wards, with seven logistics personnel allocated daily to deliver finished infusion products. After the opening of Phase II, the number of wards served increased to 40. Despite this expansion, the number of logistics personnel remained unchanged, and full-day delivery of finished infusion products for both standing and PRN orders across all wards was achieved without additional staffing. Details are provided in Table 2.

**TABLE 2:** Comparison of staffing allocation in labeling and delivery processes prior to and following transformation.

Period	Number of wards served	Personnel required for standing order labeling	Personnel required for standing order delivery
Pre-transformation	20	6	7
Post-transformation	40	4	7

This table presents count data, illustrating changes in personnel allocation and service scale before and after the renovation. No statistical comparisons were conducted; the data are provided solely for descriptive purposes.

### 3.3 Comparison of error rates prior to and following transformation

Following the smart and digital transformation of PIVAS, a substantial reduction in error rates was observed in the labeling and delivery processes, both of which had incorporated intelligent equipment. The differences in error rates between the pre- and post-transformation periods were statistically significant (*p* < 0.05), as presented in Table 3.

**TABLE 3:** Comparison of error rates in labeling and delivery processes prior to and following transformation (n = 3,000).

Period	Labeling error rate (%)	Delivery error rate (%)
Pre-transformation	0.72 ± 0.86	0.33 ± 0.48
Post-transformation	0.084 ± 0.22*	0.073 ± 0.20*
*t*	6.889	4.729
*p*	0.000	0.000

Error rate data are expressed as mean ± standard deviation (x̄ ± s). The comparison before and after modification was performed using the paired-sample t-test.

* P < 0.001 indicates significant difference compared to the pre-modification state.

## 4 Discussion

The PIVAS workflow involves a high degree of task repetition, a characteristic that makes it particularly well-suited for improvements through digitalization and intelligentization, which have demonstrated significant benefits in enhancing both operational efficiency and quality (Chen X. X. et al., 2021). In recent years, PIVAS has undergone rapid development in China. The integration of various related software, hardware, and equipment has facilitated process optimization, reduced error rates, improved efficiency, and comprehensive traceability of medication information. Unlike traditional oral medication distribution processes, the PIVAS workflow is characterized by high labor intensity, error-proneness, and unique operational logic and risk profiles, a feature that has long been overlooked in mainstream international pharmaceutical automation research. This study generated detailed daily operational data and systematically evaluated four core performance metrics of the PIVAS system: label preparation time, medication dispensing duration, staffing requirements, and error rate. Unlike previous studies conducted under stable operational conditions, this digital transformation initiative was implemented during a period of rapid service expansion: daily infusion volumes increased by approximately 50%, and hospital coverage expanded from 20 to 40 wards. This distinctive context enabled the study to further validate the scalability of intelligent PIVAS systems under surging demand, beyond merely assessing improvements in basic operational efficiency. Evidence from practical implementation suggests that intelligent and information-based transformation offers significant advantages in improving PIVAS operational efficiency, reducing errors, and alleviating labor-intensive tasks (Yang et al., 2023). It reduces the risk of manual errors such as incorrect label application, batch information confusion, quantity verification discrepancies, and departmental packaging errors. Addressing these issues at the source helps prevent downstream consequences including rework, waste, and traceability difficulties, while supporting more comprehensive information traceability and smoother clinical communication.

### 4.1 Optimizing workflow and improving management efficiency

The PIVAS workflow is more complex, involving intricate and interdependent processes when compared to conventional pharmacy operations. For personnel working in PIVAS, the tasks demand both sustained mental focus and substantial physical effort (Huang and Chen, 2017). The limitations of traditional manual procedures within PIVAS have been addressed through the implementation of smart and digital transformation initiatives. These changes have significantly optimized workflow, automated key procedures, ensured traceability across processes, and enhanced overall management efficiency. Manual tasks have been increasingly replaced by automated systems, leading to improvements in the workflows associated with labeling, packaging, and delivery. These enhancements have contributed to a reduction in physical workload and an increase in staff satisfaction. With ongoing technological advancement, the concept of “intelligent IV admixture” has received growing attention. By integrating modern technologies and utilizing automated equipment to connect various PIVAS operations through data-driven systems, a streamlined, intelligent, and highly efficient assembly line-style working model has become progressively feasible (Huang and Chen, 2017). The study demonstrates that process optimization effectively improved the intravenous medication labeling and delivery workflow. Effectiveness was evaluated across three dimensions: work efficiency, quality and safety, and number of configured personnel, using the following indicators: time per label application, infusion delivery duration per equivalent workload, labeling error rate, delivery error rate, and number of operational staff required. Following implementation, labeling time and overall delivery infusion duration were significantly reduced, and error rates in both labeling and delivery processes decreased markedly. Additionally, ward service coverage was expanded while core staffing requirements were reduced. All differences before and after optimization were statistically significant (P < 0.001), confirming that the process improvement enhanced operational efficiency and medication safety in intravenous medication dispensing and delivery, with practical value for broader clinical adoption.

### 4.2 Improving work efficiency and reducing the required number of staff positions

Prior to the transformation, under a fully manual operation model, work efficiency among personnel was inconsistent, and interference among staff during task execution contributed to low overall efficiency. Following the transformation, manual labeling was replaced by an intelligent labeling system, which significantly reduced labeling time and allowed for a reduction of two personnel. Labeling accuracy and overall efficiency were notably improved. The optimization of intelligent information collection technology further enhanced efficiency in the packaging process and enabled both PIVAS staff and ward nurses to access information on delivered finished infusion products quickly and accurately. This facilitated effective information sharing between PIVAS and clinical nursing staff, reducing the need for frequent telephone communication and allowing for more timely issue resolution. After the introduction of the intelligent delivery robot, delivery times were reduced despite an increase in workload, and the number of personnel required for delivery tasks decreased.

### 4.3 Reducing errors and improving clinical satisfaction

As the number of inpatients at the hospital increased, the workload associated with centralized preparation in PIVAS grew, resulting in greater physical demands on staff. High labor intensity, coupled with repetitive and monotonous tasks, contributed to decreased concentration and physical fatigue, thereby increasing the risk of errors due to inattentiveness. Errors in compounding have the potential to cause significant harm to patients. Implementation of the intelligent labeling system improved the working environment, reduced physical strain, and effectively mitigated manual labeling errors and infusion batch confusion. In addition, the summary drug dispensing list generated by the labeling system was clearly categorized. When compared with the pre-implementation period, errors related to unclear information on dispensing lists were reduced.

Incorrect delivery of finished infusion products to the wrong ward is a common error within PIVAS operations. Although such errors may appear minor, they can lead to serious consequences, and medication safety under manual delivery processes is not reliably ensured. The optimization of the intelligent information collection system, together with the deployment of the intelligent delivery robot, contributed to a reduction in wrong-ward deliveries and significantly enhanced operational safety. As a result of these improvements, including reduced error rates and the timely, secure delivery of finished infusion products, clinical departments reported a notable increase in satisfaction with PIVAS services (Yang et al., 2021).

### 4.4 Clinical relevance

The process improvements implemented in this study has clear practical clinical value. Shorter delivery times helps ensure timely intravenous treatment in ward areas, supporting the continuity of clinical diagnosis and treatment plans. Reduced labeling and delivery error rates lower the risk of medication errors early in the drug distribution chain, and has the potential to reduce the risk of adverse drug events downstream. Furthermore, a more efficient pharmaceutical distribution workflow reduces the time nursing staff spend on non-clinical tasks, easing workload pressure, allowing greater focus on direct patient care, with potential benefits for overall service quality and patient experience.

### 4.5 International application of smart pharmacy robots

In Europe, the Loccioni Group has developed the APOTECA series of automated pharmacy system, which have been widely adopted to improve standardization, safety, and efficiency in hospital pharmaceutical services (Westbrook et al., 2020; Lee et al., 2026). Research on these systems has provided a valuable international reference for intelligent pharmacy development. Designed specifically for high-risk formulations such as chemotherapeutic agents, the APOTECA system uses a fully enclosed negative-pressure design to reduce occupational exposure risks while ensuring sterility and precision in drug preparation (Iwamoto et al., 2017; Cho et al., 2024). Unlike the APOTECA system, which focuses exclusively on the dispensing of high-risk drugs, the intelligent PIVAS model described in this study centers on smart labeling machines and intelligent delivery robots. This configuration better reflect the practical demands of tertiary hospitals in China, where high-volume intravenous infusion workloads, diverse medication categories, and operational efficiency are primary concerns. The system supports standardized infusion label management, unmanned closed-loop drug transportation, and integration with HIS and lifecycle information management platforms. Compared with imported specialized dispensing systems such as APOTECA, domestically developed intelligent PIVAS solutions offer lower implementation costs, greater adaptability to standard infusion workflows, and better compatibility with existing hospital information systems. Together, these features reduce repetitive manual tasks and improve the overall operational efficiency of intravenous medication dispensing centers while maintaining medication safety.

### 4.6 Limitations

This study has several limitations. First, during the study period, the average daily number of infusion bags increased by approximately 1,000 compared to pre-renovation levels, substantially raising the overall workload. This present a potential confounding factor that may have independently influenced work efficiency and error rates. Because continuous daily data were not systematically collected, statistical correction through methods multiple regression or stratified analysis was not possible, which limits the interpretive the strength of causal inference regarding the intervention effect. Nevertheless, improvements in labeling time, distribution duration, and error rates were observed despite the higher workload, supporting the practical value of process optimization. Future studies should incorporate systematic daily data collection and multi-factor statistical models to better isolate the intervention effect and assess its long-term impact. In addition, as a single-center observational study conducted at a tertiary hospital, the generalizability of the findings is inherently limited. Future multicenter studies with larger samples would be needed to validate these findings.

Second, for low-frequency count indicators such as error rates, parametric tests were applied directly without the use of more appropriate models such as non-parametric tests or Poisson regression. Effect sizes and statistical power were not reported, limiting the depth of the statistical evaluation. Moreover, the outcome measures in this study focused on in-hospital workflow and operational quality, covering labeling and distribution efficiency, error rates, and service capacity. Patient level clinical outcomes, such as adverse events, adverse drug reactions, and in-hospital prognosis related to medication errors, were not assessed. This focus on process indicators alone limits the clinical relevance of the findings. Future studies should incorporate patient clinical outcomes alongside process measures to evaluate broader benefits of workflow optimization from both an operational and patient safety perspective.

In addition, due to the confidentiality of the hospital’s overall financial data, the economic analysis focused solely on short-term operational number of configured personnel, and improvements in workflow efficiency, excluding one-time upfront investments, equipment maintenance expenses, and overall facility renovation costs. The clinical benefits of this intelligent PIVAS transformation primarily include reducing medication errors, standardizing intravenous drug management, achieving full-process traceability, and minimizing manual operational risks, thereby ensuring medication safety for hospitalized patients. Future research will integrate whole-life-cycle cost data and multidimensional healthcare value metrics to conduct more rigorous health economics evaluations.

Lastly, this study used a non-randomized before-and-after controlled design, as random allocation was not feasible given the practical constraints of the research setting. The design is inherently susceptible to time-dependent confounding, which may introduce bias into the results. Factors such as learning effect, growing staff familiarity with procedures, internal management changes, and shifts in external policy during the study period may all have influenced outcomes independently of the intervention. Accordingly, the contribution of time-related confounding cannot be fully excluded, and this represents a limitation of the study. Furthermore, this intelligent transformation represents a mandatory upgrade to the PIVAS system and has been uniformly implemented across the entire hospital, making it impossible to establish an appropriate internal control group. Retaining some wards using traditional manual processes would lead to inconsistencies in the PIVAS workflow and potentially introduce security risks due to the integration of parallel systems. Given the specific timing of implementation and variations in PIVAS deployment scale, staffing configurations, and baseline workflows among hospitals, external control institutions are not available. To avoid overinterpretation of results, all findings in this study are presented as improvements at the operational level rather than absolute causal effects; additionally, the comprehensive impact of workflow digitization and service expansion has been fully considered during result interpretation. Future research using randomized controlled or concurrent control designs would help address this issue and strengthen the internal validity of findings.

### 4.7 Future outlook

With the ongoing development and expansion of smart technologies in PIVAS across China, the safety and accuracy of intelligent equipment used in PIVAS operations have been increasingly recognized by healthcare professionals. A substantial rise in demand for such equipment is anticipated in the future (Gong et al., 2024). Despite the progress achieved, there remains significant potential for further improvement in the smart and digital transformation of the hospital’s PIVAS. Several areas have been identified for enhancement: 1) The current labeling system cannot verify solvent quality or expiration dates and lacks built-in error detection, real-time tracking, or automatic error reporting. These functional limitations will be addressed in future system upgrades. 2) Currently, the delivery of finished infusion products in domestic PIVAS settings is carried out by department. Exploration of a patient-specific delivery model may offer a means of reducing the time required for secondary distribution by ward nurses. 3) The final stage of the process, from the receipt of finished infusion products prepared by PIVAS to actual infusion administration, remains disconnected from the existing digital workflow. In the future, implementation of digital bedside infusion monitoring is anticipated. Such a system could enable tracking of infusion parameters, including drip rate and duration, provide real-time professional guidance to nursing staff, and establish a closed-loop data management framework centered around PIVAS operations.

## 5 Conclusion

Through a comparative analysis of changes in work efficiency, accuracy, and information traceability in PIVAS prior to and following the implementation of intelligent equipment and information system upgrades, the smart and digital transformation was shown to optimize workflow, enhance operational efficiency, reduce human error, achieve traceability of information, and improve ward-level satisfaction with PIVAS services. These findings indicate that the transformation has contributed to notable improvements in workflow processes, management structure, and data transparency within the PIVAS of the hospital, advancing toward automation, intelligentization, and greater usability.

With increasing demand for the development of smart PIVAS systems, it is anticipated that the digital and automated transformation of key processes, including compounding, sorting, and infusion at the patient level, will continue to advance. These developments are expected to facilitate the establishment of a fully automated, closed-loop system for PIVAS operations. Strengthening automation and digitalization within PIVAS represents a critical aspect of future development and aligns with the operational model of modern smart hospitals. This direction reflects an inevitable progression in the context of an increasingly information-driven healthcare environment.

## Data Availability

The original contributions presented in the study are included in the article/Supplementary Material, further inquiries can be directed to the corresponding authors.
