# Supplementary material for: Impact of smart and digital transformation on pharmacy intravenous admixture services in a tertiary-grade A hospital
Source: Front Pharmacol. 2026 Aug 26;17:1798701. doi: 10.3389/fphar.2026.1798701 (PMC13553744; doi:10.3389/fphar.2026.1798701)
Supplement: Supplementary file 1 [file Supplementaryfile1.docx]

Operation Method of the Labeling System

1. The operator first prepares a medication dispensing summary sheet based on the drug information required for each batch as displayed by the system. The drug information on the summary sheet must fully match the infusion labels, with clearly distinguished items requiring dispensing and those not requiring dispensing.

2. Initiate intelligent labeling: During labeling, the system simultaneously performs recognition, verification, printing, and labeling. The working principle involves utilizing high-precision visual recognition technology to analyze surface characteristics such as font and color of the solvent in the recognition area, enabling real-time detection. The system instantly determines whether the solvent bag matches the solvent specified on the infusion label to be affixed. If a match is detected, the bag is conveyed via a conveyor belt to the flexible labeling station, where the labeling machine prints the label and a robotic arm applies it using a flexible negative-pressure applicator for vertical application. A covering device then smooths the infusion label to ensure even attachment. If solvent-label mismatch is detected, the system halts transmission and does not label the erroneous solvent bag.

3. After completion of labeling, the conveyor belt transports the labeled solvents to the aggregate container according to pre-set positions. Medicinal products are grouped based on formulation requirements, such as antibiotics in one group, cytotoxic drugs in another, and traditional Chinese patent medicines in a separate group. Within each group, drugs of the same manufacturer and type are grouped together. Labeling is performed in sequence according to the specific dosage requirements of each individual drug.

4. During operation, when replacing the solvent system, voice prompts will be provided. Operators should follow these prompts and check the solvent information on the main control panel to replace the corresponding solvent and continue printing labels. Solvents of different types and specifications are labeled with distinct font colors on the main control panel, with the system font color matching that of the solvent labels. For example, glucose-based infusions are displayed in red font, saline-based infusions in blue font, and glucose-sodium chloride solutions in green font, enabling staff to quickly identify and replace the solvent promptly.

5. The main control operation panel display screen shows the current infusion label information in real time, allowing for immediate viewing of the labeling details. Each label bears a unique serial number displayed in the lower-right corner, serving as a retrieval reference for subsequent verification.
